# SpheroidPicker for automated 3D cell culture manipulation using deep learning

**DOI:** 10.1038/s41598-021-94217-1

**Published:** 2021-07-20

**Authors:** Istvan Grexa, Akos Diosdi, Maria Harmati, Andras Kriston, Nikita Moshkov, Krisztina Buzas, Vilja Pietiäinen, Krisztian Koos, Peter Horvath

**Affiliations:** 1 Synthetic and Systems Biology Unit, Biological Research Centre (BRC), Eötvös Loránd Research Network (ELKH), Temesvári körút 62, Szeged, 6726 Hungary; 2 Doctoral School of Interdisciplinary Medicine, University of Szeged, Korányi fasor 10, Szeged, 6720 Hungary; 3 Doctoral School of Biology, University of Szeged, Közép fasor 52, Szeged, 6726 Hungary; 4 Single-Cell Technologies Ltd, Temesvári körút 62, Szeged, 6726 Hungary; 5 Laboratory on AI for Computational Biology, Faculty of Computer Science, HSE University, Pokrovsky Boulevard, 11, Moscow, 109028 Russia; 6 Department of Immunology, Faculty of Medicine, Faculty of Science and Informatics, University of Szeged, Szeged, 6720 Hungary; 7 Institute for Molecular Medicine Finland (FIMM), Helsinki Institute of Life Science, University of Helsinki, Tukholmankatu 8, 00014 Helsinki, Finland

**Keywords:** Assay systems, Cell delivery, Biomedical engineering, Computational platforms and environments, Data processing, Machine learning, Software

## Abstract

Recent statistics report that more than 3.7 million new cases of cancer occur in Europe yearly, and the disease accounts for approximately 20% of all deaths. High-throughput screening of cancer cell cultures has dominated the search for novel, effective anticancer therapies in the past decades. Recently, functional assays with patient-derived ex vivo 3D cell culture have gained importance for drug discovery and precision medicine. We recently evaluated the major advancements and needs for the 3D cell culture screening, and concluded that strictly standardized and robust sample preparation is the most desired development. Here we propose an artificial intelligence-guided low-cost 3D cell culture delivery system. It consists of a light microscope, a micromanipulator, a syringe pump, and a controller computer. The system performs morphology-based feature analysis on spheroids and can select uniform sized or shaped spheroids to transfer them between various sample holders. It can select the samples from standard sample holders, including Petri dishes and microwell plates, and then transfer them to a variety of holders up to 384 well plates. The device performs reliable semi- and fully automated spheroid transfer. This results in highly controlled experimental conditions and eliminates non-trivial side effects of sample variability that is a key aspect towards next-generation precision medicine.

## Introduction

In drug discovery studies, cell-based assays with cell cultures have been widely adopted. In order to understand the activity of a compound, two-dimensional (2D) model systems have mainly been used and are still in use^1^. However, it has been shown that the microenvironment of the in vivo tumor is different from the 2D monolayer cell cultures^2^. It has been also described that three-dimensional (3D) cell cultures are physiologically more relevant model systems for drug testing^3^ and cancer research because they enable the examination of drug penetration and tumor development^4,5^. One of the most common 3D cell cultures is the spheroid models where the cells aggregate, produce an extracellular matrix, consequently form a sphere-like structure^6^. Due to their 3D nature, gradients of nutrients, oxygen and pH establish over time, mimicking the physiological microenvironment of solid tumours^2,7^. Although spheroid models can bridge the gap between conventional 2D in vitro testing and animal models, it is still a challenge to use these 3D cell cultures in a high-throughput system. The lack of a unified protocol for creating spheroids results in varying shapes with huge morphological heterogeneity^8^ that is not ideal for clinical studies. However, it was recognized more than a decade ago that uniform morphology, microenvironment and cellular physiology of spheroids are mandatory to ensure reliability of single assays and multiple independent assay series as well^9^. In most cases pre-selecting spheroids by their morphology is a very time-consuming and inconsistent process because it requires human assistance. Moreover, the expert selects the spheroids by the naked eye or in the case of smaller samples (under 500 µm in diameter) under a simple microscope, which can not guarantee uniformity.

Spheroids derived from cell lines and patients/donors will result in different shapes and sizes that can make the selection difficult, especially by naked eye. Therefore, detection and segmentation must be precise to achieve highly accurate features. Previous selection techniques are based on subjective decisions of experts, who specified the optimal conditions, such as area or perimeter^10^. Unfortunately, the imaging conditions can strongly vary, such as light, illumination, density of the medium, or the shape of the plates that can affect the expert’s decision. Spheroids should be detected and segmented on label-free brightfield microscopy images. Methods that handle such images are based on classical analysis methods like thresholding or watershed^10,11^. Most recently, deep learning object detection and segmentation methods have become popular since they provide more accurate results, and do not require fine-tuning even if imaging conditions change^12^.

Existing commercial devices are able to automate various parts of cell culture handling, for example, automatic spheroid generation with SphericalPlate 5D^13^. These regulate the spheroid size, which is just one step for strandization. Most of the systems support liquid treatment but not selective transfer. Devices created for plate to plate transferring^14^ do not use advanced cell detection methods and always expect one cluster or spheroid per plate. Methods used for detection are threshold-based methods, which can lead to inappropriate feature calculation, e.g. regarding the size of the cell culture. These methods usually utilize machine learning only for cell classification, but not for segmentation.

**Figure 1:**
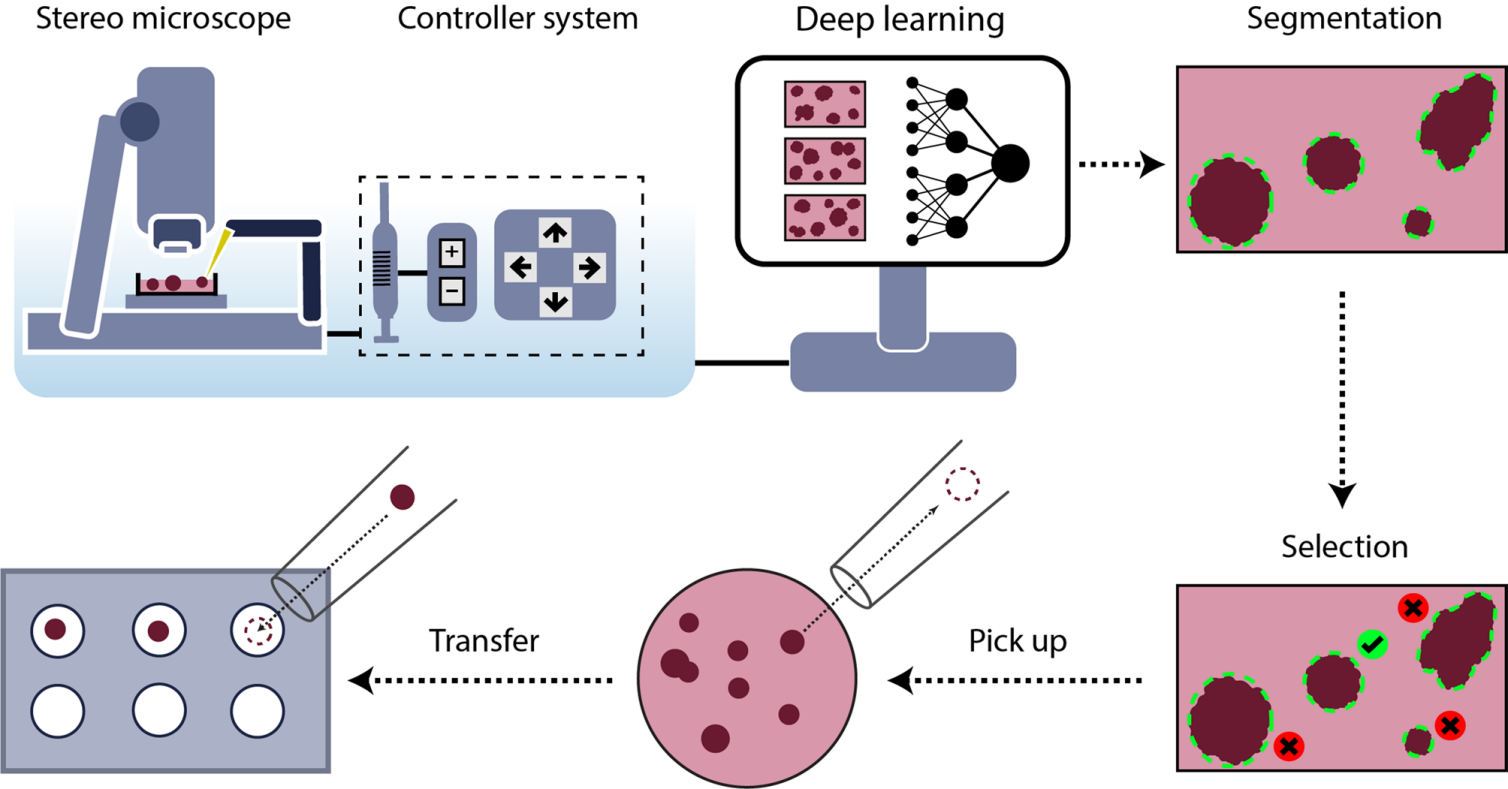
Schematic representation of the SpheroidPicker. The system includes a stereo microscope, syringe, stage, and manipulator controller. The automatic screening function results in brightfield images of the spheroids. The segmentation and feature extraction steps are based on a deep learning model. After the selection of the spheroids, the spheroid picker automatically transfers the spheroids into the target plate.

In this work, we propose a new solution to work with spheroids. We designed and built a robotized microscope that can support or fully replace experts by automatically selecting and transferring 3D cell cultures for further experiments (Fig. 1). We developed a fast and accurate deep learning-based framework to detect and segment spheroids and integrated it into the machine’s controller software. We have created a unique image database of annotated spheroids and used it for training deep learning models. We show that our models can detect and segment spheroids with high reliability. Using such accurate segmentation, the algorithm is able to extract features robustly and—based on the user’s criteria—decide which spheroids to manipulate. This system was developed to automate one of the most time-consuming processes during sample preparation, the pre-selection phase, where accuracy is crucial.

## Materials and methods

### Components of the system

The system is built on a stereomicroscope (Leica S9i, Germany) with a large working distance to fit the pipette between the sample and the objective. This microscope has an integrated 10MP CMOS camera directly connected to the controller PC via USB port. The motorized stage (Marzhauser, Germany) has a 100 × 150 mm movement area. We designed a 3D printed plate holder that is compatible with the most common microwell plates, including 24-, 96-, or 384-well plates (Fig. 3c,d). A two-axis micromanipulator is installed next to the microscope and can be controlled at a micrometer precision. A custom-designed stepper motor-driven syringe pump ensures high precision (3 µl) fluid movement. The whole system is small, thus mobile, and can be installed under a sterile hood.

The software performs four major operational steps (1) entire hardware control, (2) automated imaging, (3) spheroid detection using a trained Mask R-CNN^15^ deep learning framework (described later), and (4) an easy to use graphical user interface to define custom spheroid selection and transfer criteria.

### Custom micromanipulator and syringe pump

The custom micromanipulator is designed and built based on the openbuilds^16^ linear actuators. The mainframe is built from 2080 (C-beam) aluminum extrusion. An M8 threaded rod with 2 mm pitch moves a four-wheel carriage. A Nema 17 two-phase bipolar stepper motor is used to directly rotate the threaded rod which provides enough torque and holding force. Each actuator has two endstops that are simple switches, and when they are hit with the carriage they help to avoid collision. These also guarantee the precise initialization at startup by setting these positions as the origin. Two of these actuators are assembled at 90°, one of them provides axial positioning, the other sets the height of the pipette. The pipette is a glass capillary rod that is inserted into a capillary holder and attached to a 3D printed part.

The custom syringe pump is a simplified linear actuator that is moved by a stepper motor. It consists of 3D printed parts. Sterile or disposable syringes can be used to ensure the sterility of the system. The syringe is connected to the capillary holder with a silicone tube. This tube has to be filled with liquid (without air) beforehand to ensure precise flow. The parameters of the capillary are 1 mm OD, 0.6 mm ID, and 40 mm in length. The hardware is controlled with an Arduino Mega 2560 microcontroller combined with “ramps 1.4” shield. This shield provides routes and pinouts from the Arduino which makes it easy to connect stepper motors, drivers, and high power.

The Marlin open-source CNC controller software is used as firmware. It operates with G-code commands that are sent via serial port. The framework has many additional features besides simple hardware driving, such as speed, acceleration, and jerk control.

### 3D printing

Parts were 3D printed with an unmodified Original Prusa i3 MK3s, which is an FDM cartesian type 3D printer. It melts the spooled thermoplastic with a hot end on the printhead and is deposited on growing work. The head is moved with a small computer control. The plate holders were created with PLA, printed with 60 °C bed temperature and 210 °C hotend temperature. The parts of the manipulator were printed with PETG 90 °C bed temperature and 245 °C hotend temperature. The models were designed in Autodesk Inventor and sliced with PrusaSlicer. The printer has the default installed with a brass 0.4 mm hole diameter nozzle, and we used 0.15 mm layer height.

### Software

**Figure 2:**
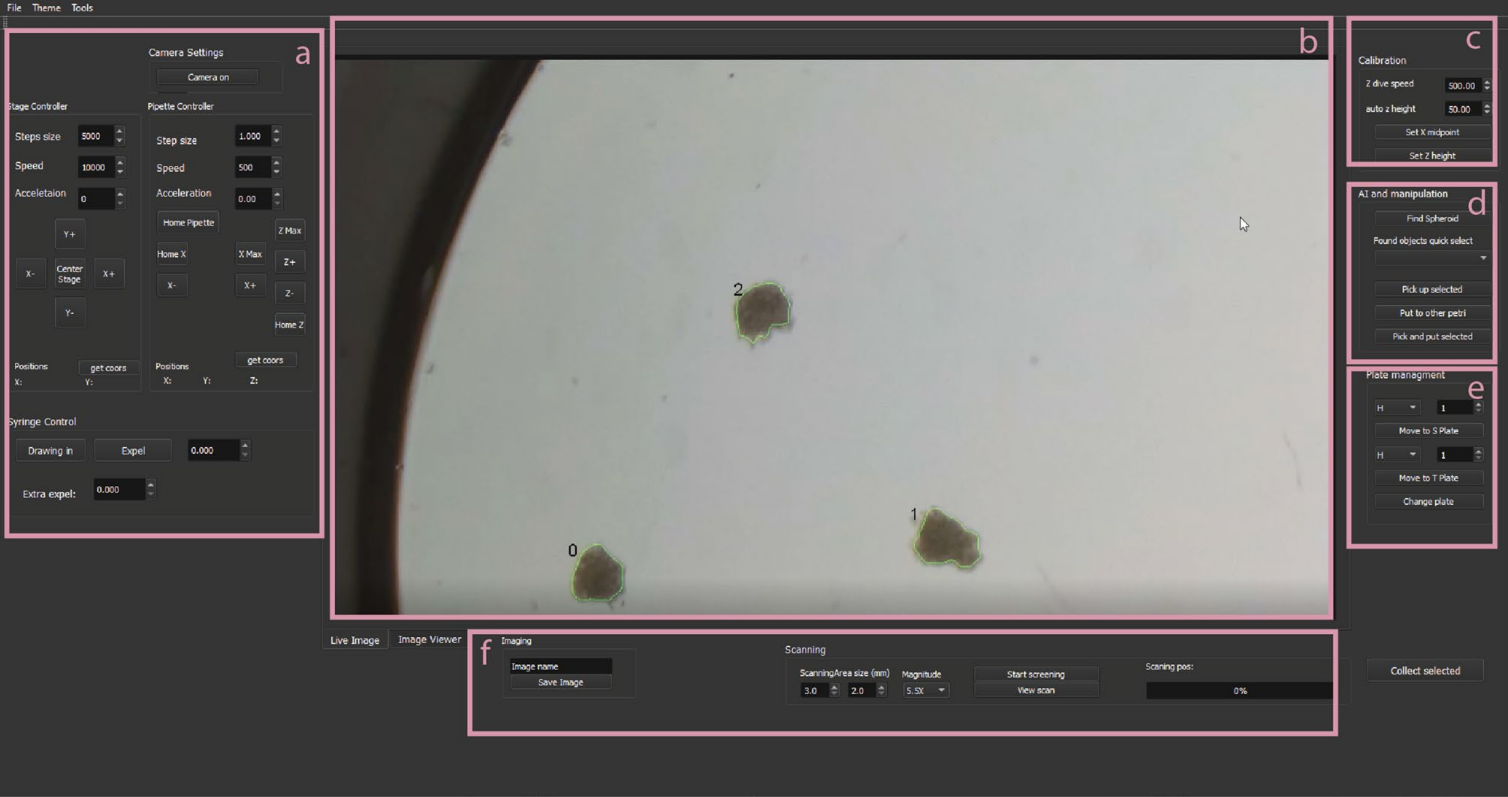
The main window of the software. The user can access the hardware control, imaging, and spheroid detection system. It can be used for spheroid manipulation, plate management, and scanning. (**a**) Hardware control tools, e.g. pipette or stage movement, or syringe dosage. (**b**) live or segmented image. (**c**) Picker calibration tools. (**d**) Semi-and fully automatic spheroid manipulation tools. (**e**) Buttons for a quick jump to wells. (**f**) scanning tools.

The main controller software is written in C++. It has an easy to use GUI implemented in the QT framework (Fig. 2). The goal of the software is to operate all the hardware components through serial connections that are the digital camera, stage, and micro-pipetting system.

The system has to be calibrated before the first use to be able to move the pipette based on image coordinates. The process can be initiated from the software and consists of the following steps. First, the user puts a well plate in the holder and safely moves the pipette under the objective. Then the pipette tip should be moved as close to the surface of a well as possible to determine the lowest height without crashing into the plate. Finally, the tip should be moved to the center of the image and register the coordinates in the software. When calibrated, the system works with every supported plate. The process should take less than 5 min.

The software includes a Mask R-CNN deep learning model trained to segment spheroids. Input images are downscaled to 1024 × 1024 pixels by keeping the aspect ratio and zero-padding. The model is developed with TensorFlow/Keras libraries, the images are handled with OpenCV libraries. With a mid-range GPU (NVIDIA 1050 Ti 4 Gb GDDR5, 768 CUDA cores @1430 MHz) the inference takes about 1 s per image. After segmentation, the main features of the objects are calculated, including area, perimeter, circularity, mass center, and stored stage coordinates at the imaging position.

The software can scan the supported plates and select target spheroids that satisfy the requested properties. When the scanning finishes, the transfer protocol can be initiated. The stage centers on one of the selected objects in the camera image, the X-axis (effector) moves the pipette above it, and Z-axis lowers close to the well surface. A negative flow is applied with a syringe pump that soaks up about 3–4 μl fluid with the target spheroid. Afterward, the pipette is lifted and the stage moves the target plate and well under the objective. Then the pipette is lowered and the spheroid is pushed out to finish the transfer. Finally, the system either starts over the process with the next spheroid or pulls out the pipette of the field of view to finish the session.

### Cell cultures

The T-47D human breast cancer cell line was obtained from the American Type Culture Collection (ATCC) and cultured in RPMI-1640 (Lonza) supplemented by 10% FBS (EuroClone) and 0.2 unit/ml insulin (Gibco, Thermo Fisher Scientific). Huh-7D12 human hepatocellular carcinoma cells were purchased from the European Collection of Authenticated Cell Cultures (ECACC) and cultured in DMEM (Lonza) supplemented by 10% FBS and 1% l-glutamine (Lonza). 5-8F human nasopharyngeal carcinoma cell line was kindly provided by Ji Ming Wang (National Cancer Institute-Frederick, Frederick, MD, USA) and cultured in DMEM-F12 (Lonza) supplemented by 10% FBS. A549 human lung carcinoma cells from the cellbank of the Biological Research Centre were cultured in RPMI-1640 supplemented by 10% FBS. All media contained 1% Penicillin–Streptomycin–Amphotericin B mixture (Lonza) as well. Cell cultures were maintained at 37 °C and 5% CO_2_ in a humidified incubator.

### Spheroid generation and fixation

Multicellular spheroids were created by Sphericalplate 5D (Kugelmeiers) based on the manufacturer’s instructions. To seed 750 cells for each microwell/spheroid, we applied 5.625 × 10^5^ cells in 2 ml medium per well. The spheroidization time was 7 days for the T-47D, 5-8F, and A549 cell lines, and 4 days for the Huh-7D12 cells to reach similar spheroid diameters ranging from 200 to 250 µm. Culture media were changed every other day during the incubation time. After the spheroids developed, they were washed twice with DPBS and fixed with 4% paraformaldehyde for 1 h at room temperature. Finally, the spheroids were washed twice with DPBS and stored at 4 °C in DPBS. This technique is suitable to produce a maximum of 750 spheroids in one well, and generation of fixed spheroids were repeated 2 times for each cell line.

### Morphological and viability analysis of spheroids

Living T-47D, 5-8F and Huh-7D12 spheroids generated in Sphericalplate 5D were used to test the effects of spheroid picking on morphology and viability. Spheroidisation time was 5, 1 and 4 days, respectively based on the characteristic duplication time of the cells, and the seeded cell numbers were 750 or 1000 cells/microwell. For the long-term morphological and viability experiments, we acquired both brightfield and fluorescence images of the spheroids at 0, 24, and 48 h after we transferred them into a 96-well U-bottom, cell repellent microplates (Greiner Bio-One). In case of short-term viability, the spheroids were stained, then imaged directly before and after transferring to compare the immediate effect of the picking. For comparison, we also put spheroids manually on the same plate. All experiments were repeated on 3 different cell lines with 10 spheroids/groups.

For the fluorescence images, a mixture of three dyes, calcein AM (5 µM, Invitrogen, Thermo Fisher Scientific), ethidium-homodimer-1 (Eth-D1, 4 µM, Invitrogen, Thermo Fisher Scientific) and Hoechst 33342 (1 µg/ml, Sigma-Aldrich) were used to stain spheroids. Dye solutions were prepared in serum-free DMEM immediately before use and incubated on the spheroids for 20 min at 37 °C. Without washing, the spheroids were imaged and the mean intensity of the Eth-D1 stain was measured in the whole area of the spheroids (n ≥ 5). Brightfield images were analysed by the AnaSP software^17^ to quantify the morphological parameters of the spheroids, e.g. equivalent diameter, solidity, area or volume.

### Image acquisition of living spheroids

For the morphological and viability images, the Leica TCS SP8 microscope (Leica Microsystems) was used. The fluorescence images were captured with the DigitalCameraDFC in 1392 × 1040 pixel resolution with 0.775751 µm pixel size. Images were taken with a HC PL FLUOTAR 5×/0.15 dry detection objective and the exposure time was adjusted for each channel separately (brightfield 35 ms, FITC 15 ms, TXR 125 ms, and DAPI 250 ms).

### Statistical analyses

Statistical analyses were performed by R studio. For the statistical analyses of the morphological and intensity results, one way ANOVA comparison was performed. Significance level was set to ɑ = 0.05 with a 95% confidence interval, and p-values were adjusted to account for multiple comparison.

### Data generation

We have acquired 981 brightfield images of spheroids in very different illumination conditions. These images (1920 × 1080, 24 bit RGB) were annotated by expert biologists using ImageJ^18^ to generate mask images as ground truth data for the training of the model. Finally, we had 1871 annotated objects. We divided these images into 3 sets: 70% for training, 15% for validation, and the rest 15% is used for testing.

### Segmentation methods

Precise image segmentation is the foremost important step of our spheroid selection pipeline. We measured the precision and sensitivity of four different widely used segmentation methods. Classical Otsu thresholding and watershed-based segmentation were benchmarked using the CellProfiler framework (CP)^11^. Furthermore, U-Net^19^ and Mask R-CNN^15^ deep learning techniques were adapted to the problem. CellProfiler^11^ is a widely-used bioimage analysis software including methods to segment, measure, and analyze cellular compartments. We have created 2 pipelines: for primary object detection one uses the Otsu thresholding method and the other uses the watershed method. Both pipelines have additional filters to remove the objects which have a too small or too large area or have an irregular shape. Important to note that, the CP parameters were set to get the best results on all the test images, not for images individually.

The U-Net neural network is designed for biomedical image analysis^19^ and in total has 23 convolutional layers. It is designed for semantic segmentation which does not handle overlapping objects. However, since spheroids usually do not touch or overlap, we consider the network appropriate for this problem. We trained U-Net for 100 epochs with learning rate (LR) = $3\times {{10}^{-5}}$ using Adam optimizer. The model was trained on a PC with Intel Xeon CPU E5-2620 v4 @ 2.10 GHz CPU, Nvidia Titan Xp 12 GB VRAM GDDR5x (founders edition, reference card), 3840 CUDA cores @1544 MHz with Pascal architecture, 32 GB DDR4 of RAM.

Mask R-CNN is a general framework for the instance segmentation task^20^. We trained Mask R-CNN with ResNet101 backbone initialized with COCO weights. The training consisted of 4 stages and 100 epochs in total, where different layers were updated and the learning rate (LR) was changed. In the first 4 epochs, we updated every layer in the ResNet with LR = $5\times {{10}^{-4}}$. In epochs 5–7 only 5th and above layers were updated with LR = $3\times {{10}^{-5}}$, and finally only the network head layers with LR = $1\times {{10}^{-7}}$. LR values were empirically selected based on the loss function values. The training, performed on the same system as U-Net.

## Results

**Figure 3:**
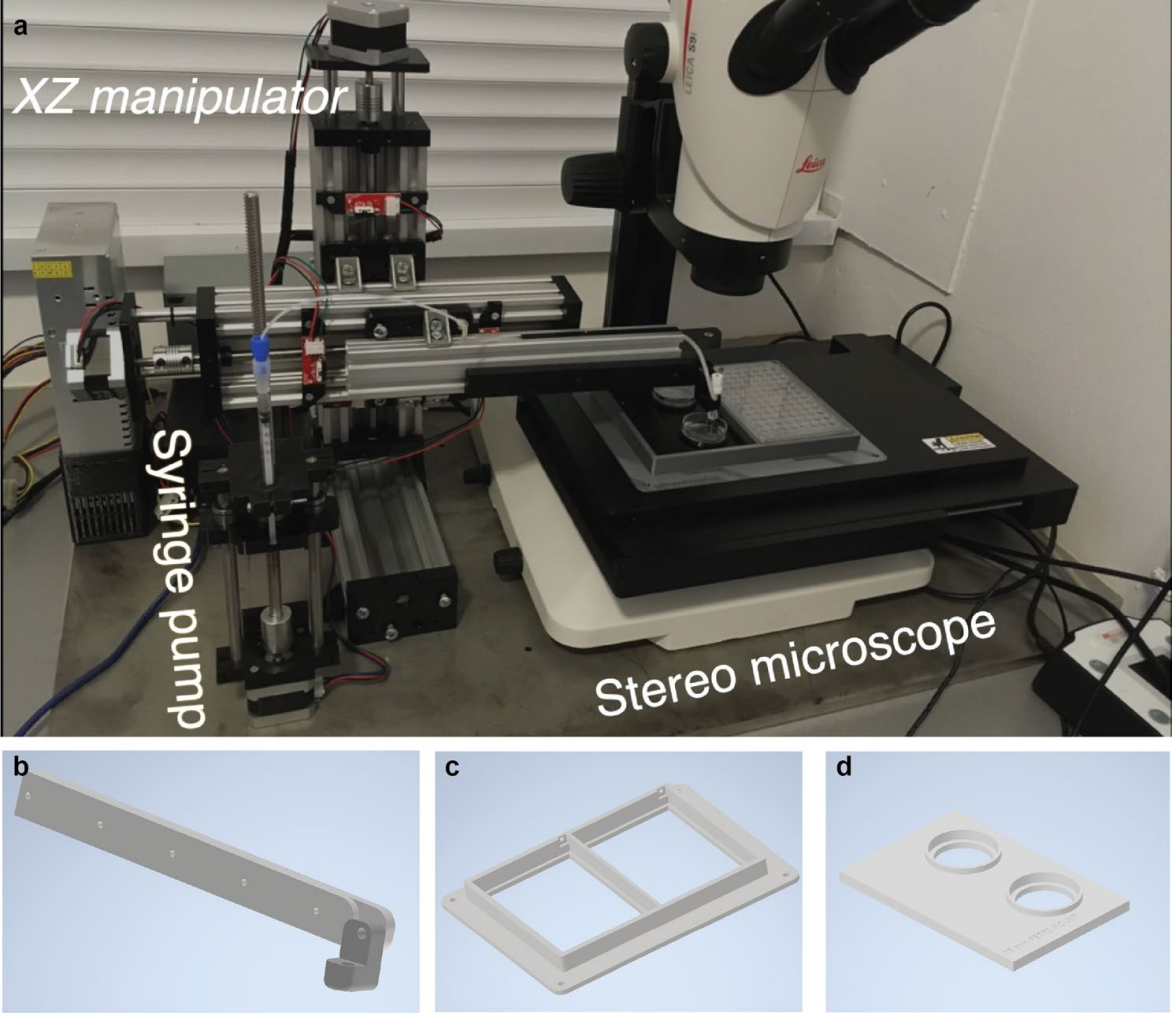
(**a**) SpheroidPicker prototype. The main components are shown, which are the manipulator, the syringe pump, and a stereo microscope. (**b**) The 3D printed element that fixes the capillary holder to the linear actuators. (**c**) 3D model of the custom-made plate-mounting system that is compatible with the most common 24-, 96-, 384-well plates. The source and target plate can be placed next to each other. (**d**) Model of a petri dish holder which can be inserted into the 96 -well plate holder.

Here, we describe how we developed and built hardware and software solutions (“Materials and methods”) to automate the spheroid handling process between various well plates (Fig. 3). The main parts of the hardware are a stereo microscope, a micromanipulator, a syringe pump, and a computer (Fig. 1).

The large field of view of the microscope allows for the efficient and fast screening of the samples. Various well plates and Petri dishes are supported so that wells or regions can be selected or excluded from analysis. The movement area of the motorized stage is large enough to place the source and target plate next to each other, and switch between them easily. The micromanipulator moves a glass capillary rod that is used for transferring the spheroids. Its axes can be configured to move it based on image coordinates. The capillary is further attached to an automated water-based syringe system. Except for the microscope, the stage, and the camera, all the hardware elements are custom built. The components are controlled from a single, custom software running on a computer. The device creates a standardized protocol for spheroid handling which is absolutely necessary for drug screening experiments.

A key feature of the system is the automatic spheroid selection capability. The operator can specify morphological properties that are required for the selected objects, such as a size range or circularity value. For the calculation of these values, a reliable image segmentation method is needed. We compared four segmentation methods which are Otsu thresholding, watershed algorithm, U-Net, and Mask R-CNN. In general classical image analysis methods could be used for the segmentation task, however, they are rather sensitive to changes in imaging conditions such as light, medium density, object transparency, shape diversity, and touching objects. Another disadvantage of these methods is that the user has to reconfigure parameters at every experiment, which can lead to loss of morphological features or getting false results. On the other hand, deep learning methods have gained attention during the last few years due to their learning capabilities and robustness. We show that our deep learning models can reliably predict spheroids without reparameterization in every condition even if the image is out of focus.

**Figure 4:**
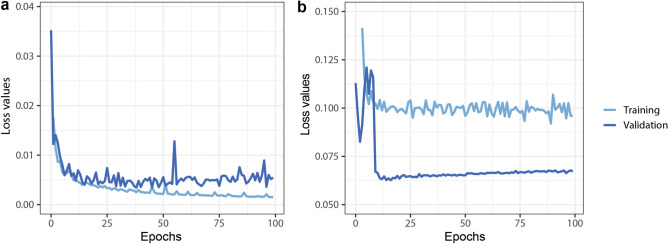
Deep learning training and validation loss values through 100 epoch training. (**a**) U-net, (**b**) Mask R-CNN. Both Neural networks are converged, with small training and validation loss values.

We trained a Mask R-CNN and U-net deep learning framework to segment the spheroids. The learning curves are shown in Fig. 4. We used the multitask loss function defined in the Mask R-CNN architecture which combines the classification loss, localization, and segmentation. For U-net we used binary cross-entropy loss functions.

**Figure 5:**
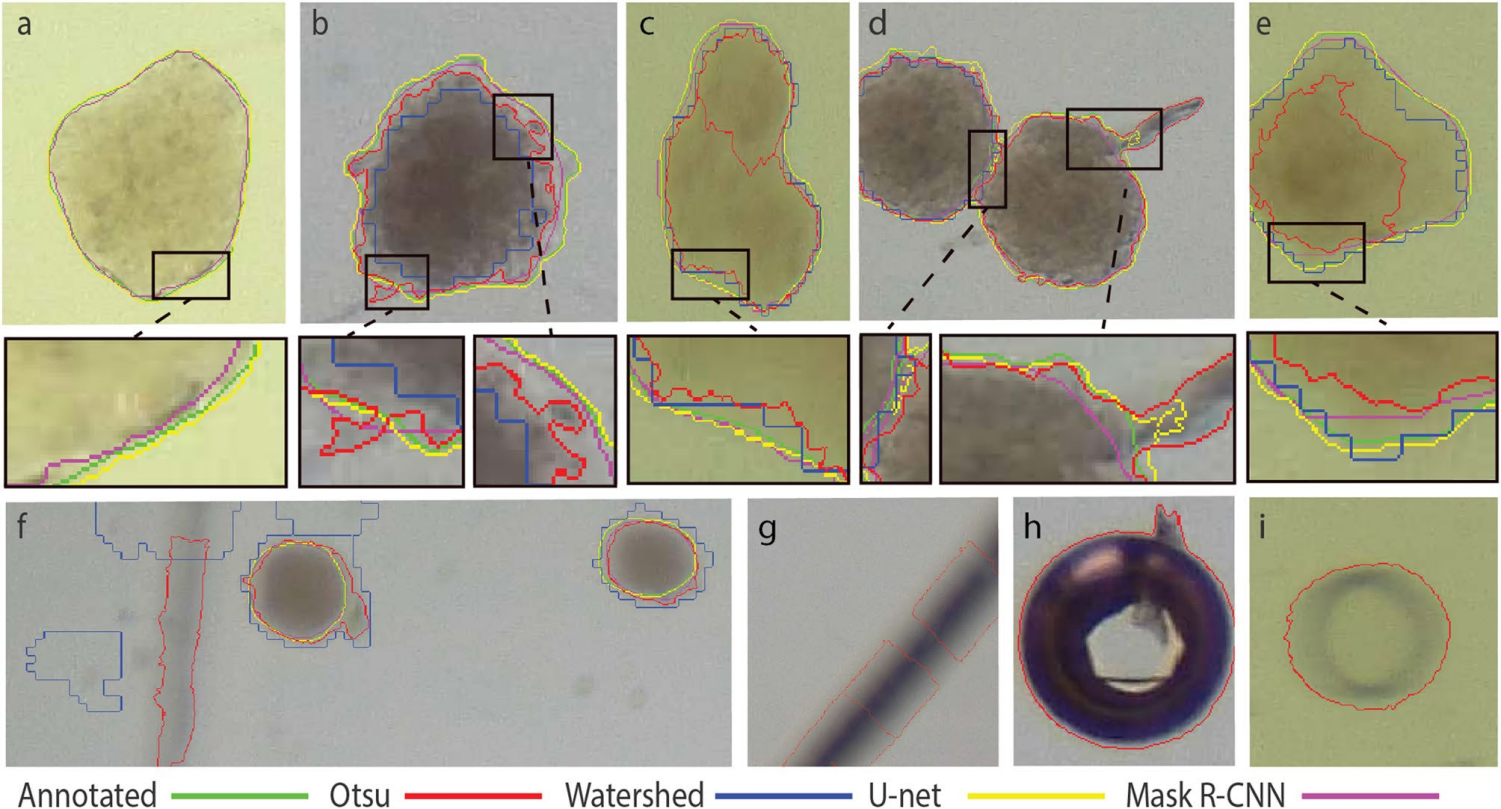
Brightfield images of spheroids with various shapes and sizes. Images contain results of 4 different segmentation methods and the annotated ground truth with color codes: green—ground truth; red: Otsu threshold; blue: watershed; yellow: U-net; magenta—Mask R-CNN. (**a–e**) Selected results and enlarged regions. In (**a**), only deep learning methods could find the spheroid. In (**d**), we can observe that the U-net is not able to completely differentiate two very close spheroids. In (**c**), thresholding identifies one spheroid as two because of the intensity changes. (**f–i**) Artifacts made by thresholding and watershed. These are usually out of focus spheroids, borders of the plates, plastic errors in plates, or air bubbles.

**Figure 6:**
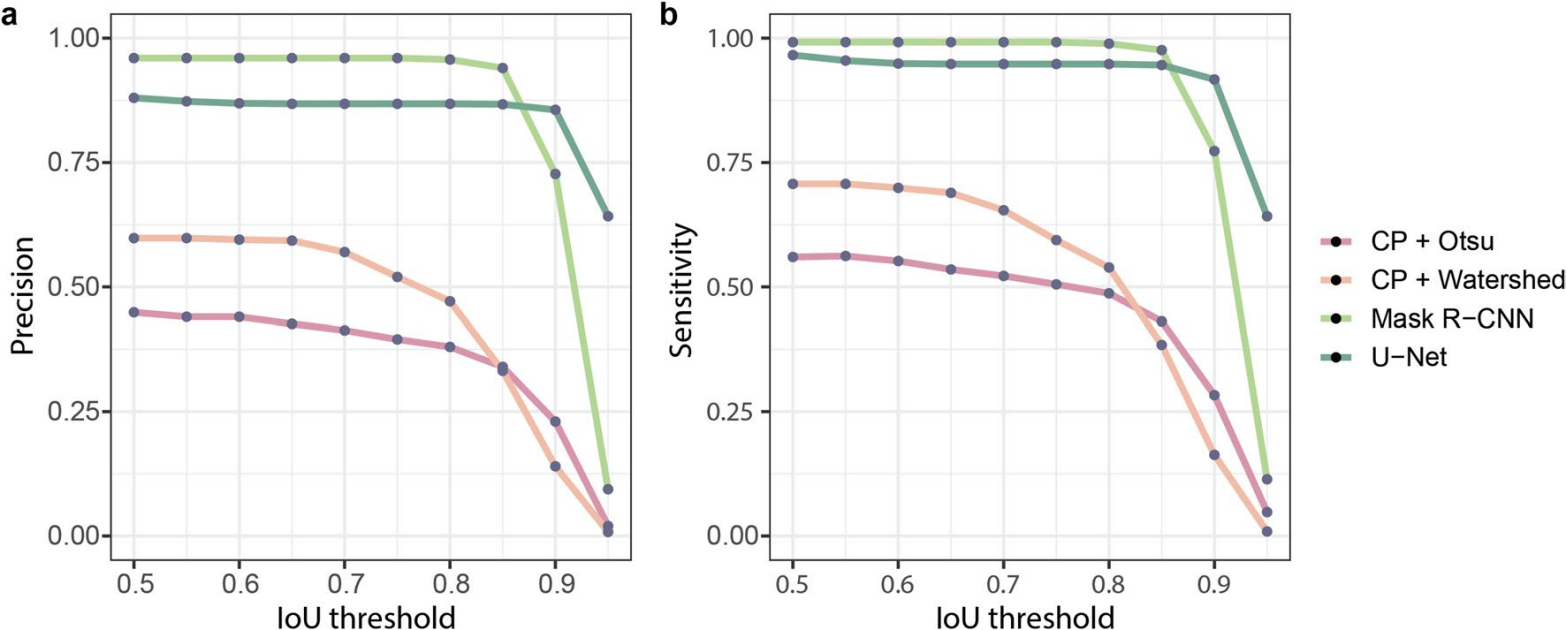
Comparison of the segmentation methods. (**a**) Average precision, (**b**) sensitivity scores, at the different intersections over union thresholds.

The model's performance was measured on a test set that consisted of 153 images. We selected in both Mask R-CNN and U-net the model with the lowest validation values. First, we analyzed the quality of the 4 segmentation methods. The results of the classical methods and the deep learning model were compared to the expert annotations (Fig. 5). We concluded that one classical segmentation pipeline cannot give reliable results in different experimental conditions. Although some spheroids are recognized remarkably well, in most cases it fails to find precisely spheroids (Fig. 5b). Deep learning methods give very small false results, and contours are almost pixel-wise precise. We can observe some cases where the U-net is not able to completely differentiate two very close spheroids (Fig. 5d). However, the contours produced by U-net are usually closer to the annotated, than Mask R-CNN (Fig. 5a), which is also confirmed by precision comparison (Fig. 6).

We compared the described segmentation methods by measuring the average precision (P) and sensitivity (S) scores (Eq. 1) on different IoU threshold values (Fig. 6). Classical segmentation methods give us worse AP and S values, because of the changing light conditions and heterogeneous shapes of spheroids. Regarding deep learning methods, Mask R-CNN with IoU value between 0.5 and 0.85 performs better in terms of average precision and sensitivity scores than U-Net. This means that Mask R-CNN detects the objects more reliably, but segments them less accurately. We can see that U-Net results in better AP and S on higher 0.9–0.95 IoU values, which means it is better for pixelwise object segmentation. As we use those results for object manipulation too, accurate object detection is more important, than slightly more accurate pixel wise segmentation.

1 $$P= \frac{True \; Positive}{True \; Positive + False \; positive+False \; Negative}, S = \frac{True \; Positive}{Positive}.$$

### Morphological and viability tests

**Figure 7:**
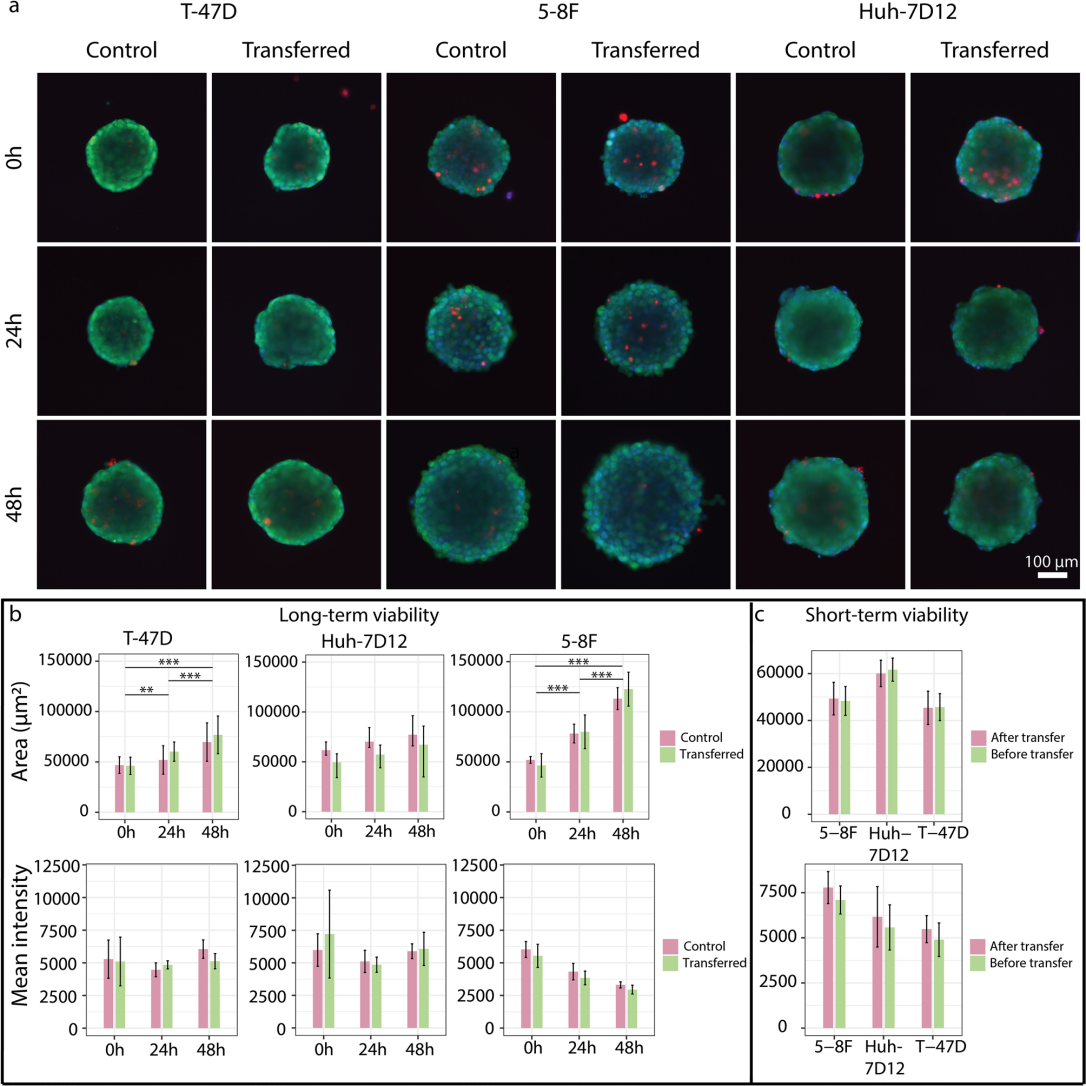
Short- and long-term viability assay of spheroids. (**a**) Fluorescence images of the control and transferred spheroids at 0–24–48 h, stained by calcein AM, ethidium homodimer-1 and Hoechst 33342. (**b**) The area and the mean intensity of EthD-1 stain were visualized for the long-term viability where spheroids were stained and imaged at 0–24–48 h. The significance was visualized only for the transferred groups. (**c**) The area and the mean intensity of EthD-1 stain were visualized for the short-term viability where each spheroid was stained, then imaged before and after transferring. Control spheroids were used in both experiments. The analysis of spheroids was performed by using the AnaSP software and the number of spheroids in each group was n ≥ 5. In the images, the scale bar represents 100 µm.

We have compared living spheroids generated from 3 different cell lines. In the short-term viability assay, spheroids were investigated before and after picking through fluorescent staining by calcein AM, ethidium homodimer-1 and Hoechst 33342 conventionally used to test viability of the 3D cell cultures (Fig. 7a). We could not find statistically significant differences when we compared the morphology and the mean intensity of the EthD-1 signal in the area of spheroids before and after transferring them between plates (Fig. 7c). We have also checked the spheroid’s long-term viability. We compared the staining and morphology of the picked spheroids after 0–24-48 h to a group of unpicked ones. We couldn’t measure any significant degradation effect of the transfer compared to the control. Furthermore, the size of the spheroids increased significantly in case of T-47D and 5-8F spheroids. These results suggest that spheroids retained proliferation capacity as well (Fig. 7a,b). Analysing the brightfield images of the SP8 by the AnaSP software developed for automatic analysis of morphological parameters of spheroids, we were able to quantify several features, for instance, area, circularity, equivalent diameter, solidity, sphericity, volume of the spheroids that we included in Supplementary Table 1.

### Quality assessment

We identified and measured different types of errors the system makes. The first type is when the target object is not sucked properly into the capillary or it falls out before getting to the target well (pick up error). The second type is when the spheroid is present in the capillary but the system is unable to put it in the target well, usually due to adherence (expel error). In the third error type the system picks up two or more spheroids, which happens when these are very close to each other and one of them is the target (double pick).

**Figure 8:**
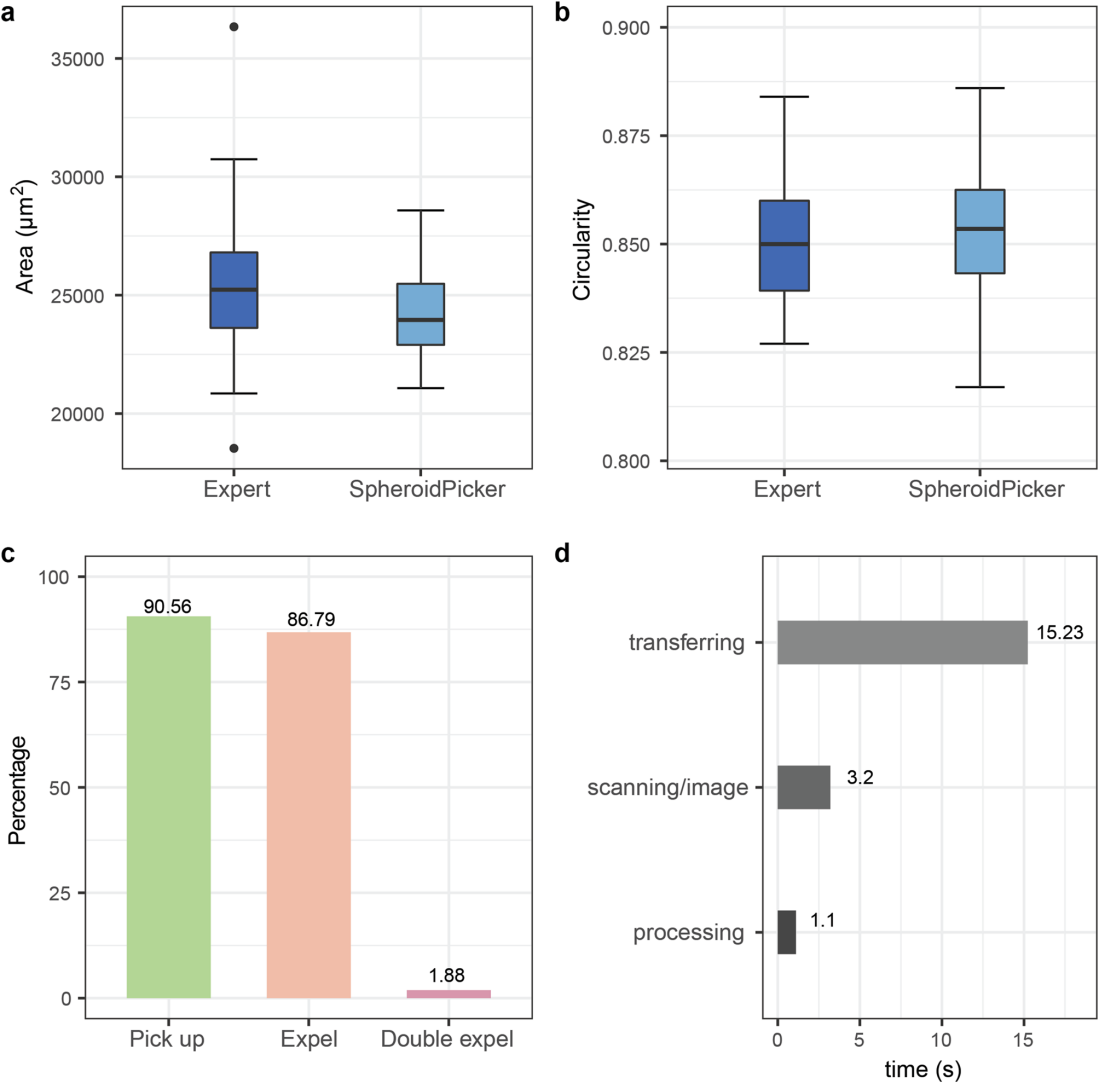
Benchmarks of the SpheroidPicker. (**a**,**b**) Precision comparison between the expert and the SpheroidPicker. The expert selected spheroids with similar size range under the stereomicroscope. For the SpheroidPicker, 21,000–29,000 µm^2^ area range and higher than 0.815 circularity were used as selection criteria. (**c**) Statistics of SpheroidPicker’s reliability. (**d**) Run times of the SpheroidPicker’s tasks.

The spheroid transferring capabilities of the system were tested by moving them one by one from a source 96 well plate to a target plate of the same type. The selection criteria was an area range between 21,000 and 29,000 µm^2^ and a minimum of 0.815 circularity. A minimum circularity criteria ensures that the selected object has a rounded shape. First, we performed this experiment such that the outcome was evaluated right after every transfer and possible issues were fixed (e.g. removed spheroids that stuck in the capillary) to be able to continue. In general from 28 attempts, 26 spheroids were successfully picked up, 25 were transferred properly, and always one object was picked up, which leads to a success rate of 89% (Fig. 8c). Each experiment was repeated 3 times. Next, we let the system fully automatically select and transfer spheroids between the plates. The Picker scanned a user-defined area of the sample and analyzed it. Out of 30 attempts, 24 were successful which leads to an 80% success rate.

We compared the selection capability of the Spheroid Pickers to the manual process of our expert with the following experiment. First, we asked the expert to manually select preferably circular spheroids with an area in the range of 21,000–29,000 µm^2^ (preferably 25,000 µm^2^, or with 178.41 µm diameter) under the microscope with a regular laboratory pipette, and transfer them separately to the wells of a 96-well plate. Then we set the same requirements in the software and transferred spheroids using it in the order it offered them. Both the expert and device transferred 42–42 spheroids. Afterward, we used our system to determine the size and circularity properties of the manually transferred objects. The average area was 25,347.2 ± 3226.3 µm^2^ in the manual case and 24,222.6 ± 1957.7 µm^2^ in the automatic, while the circularity value was 0.8504 ± 0.0147 and 0.8527 ± 0.0150, respectively (Fig. 8a,b). We also measured the average time of different processes of the picker with the software: transferring, scanning and image processing (Fig. 8d).

## Discussion

We have designed and built an automated microscopy system that is capable of selecting and transferring spheroids. The selection and the feature extraction of the spheroids are based on deep learning methods. We created to our knowledge the first annotated image database to train models for spheroid segmentation. The image processing methods used for the detection and selection are reliable, and we have shown that it can outperform human selection skills. The machine can perform semi- or fully-automatic transferring of spheroids to any predefined well plate. The greatest advantage is that it helps in selecting similar spheroids to standardize the pre-selection phase and thus make it easier and more robust. Comparing the expert and the SpheroidPicker selection capability shows that the manual approach has 1.648 higher standard deviation in terms of area, and thus our system is more reliable when specific features are required. However, the software can be improved by parallelizing scanning and image analysis tasks to speed up the process. Pick up failures and double pickups could be avoided or corrected with object detection methods, thus getting better reliability. Finally, comparing the spheroid's viability and morphology before and after picking shows that the SpheroidPicker does not affect the viability and there was no degrading effect during transfer compared to the control groups.

There are existing devices on the market that can perform transfer automatically like Cell Handler, and Cellcelector^14,21^, however these are more complicated systems and designed for general purpose. SpheroidPicker designed for especially working with 3D cell cultures. As the system provides a fast and reliable selection and transferring of 3D objects, it can be widely used in basic research and clinical studies as well for high-throughput assays. Although in this study, carcinoma spheroids were used, SpheroidPicker may be suitable to select 3D co-cultures as well, including organoids, which model system is able to mimic the intra‐ and inter‐culture heterogeneity seen in vivo^22^. This would further broaden the potential use of the system, for instance to translational applications, such as for functional precision medicine utilizing patient derived cells grown in 3D for personalized drug testing^23^.

## Conclusion

In summary, we have shown that building a microscopy system that combines AI and robotic techniques could improve efficiency in laboratory work with spheroids. To our knowledge this is the first deep learning-based manipulator robot that is designed for spheroid transfer. It uses advanced deep learning-based methods for detection and segmentation. Compared to other devices this machine costs significantly less. The spheroid picker requires a small space since the main components are a stereo microscope with a stage and the custom manipulator ensuring to set up and use under sterile hood. In this study, we also proved that spheroid picking does not affect spheroid morphology, viability and proliferation capabilities. Therefore, we suggest SpheroidPicker to select 3D cultures for further experiments, including drug tests in order to ensure reliability and reproducibility and also reduce the costs and time requirements of the research before a potential preclinical study.

## Supplementary Information

Supplementary Table 1.

## Data Availability

Annotated data is available on request. Additional video about the device is available at: https://www.youtube.com/watch?v=_3JWILPxqiM; software data is available on request.
