# Supplementary material for: SpheroidPicker for automated 3D cell culture manipulation using deep learning
Source: Sci Rep. 2021 Jul 20;11:14813. doi: 10.1038/s41598-021-94217-1 (PMC8292460; doi:10.1038/s41598-021-94217-1)
Supplement: Supplementary file 1 — Supplementary Table 1. [file 41598_2021_94217_MOESM1_ESM.docx]

| Long-term viability test |  |  |  |  |  |  |
| --- | --- | --- | --- | --- | --- | --- |
| T-47D | Avarage values | | | Deviance values | | |
|  | 0h | 24h | 48h | 0h | 24h | 48h |
| Area non-picked | 46825.98 | 51943.21 | 69671.14 | 8210.712 | 14126.76 | 19051.66 |
| Area picked | 46135.6 | 60173.28 | 76821.9 | 8575.558 | 9531.956 | 18681.64 |
| Circularity non-picked | 0.926341 | 0.939342 | 0.958388 | 0.025535 | 0.027641 | 0.013688 |
| Circularity picked | 0.950248 | 0.953604 | 0.942686 | 0.017586 | 0.022611 | 0.030435 |
| Compactness non-picked | 0.83643 | 0.863719 | 0.91517 | 0.064266 | 0.063548 | 0.017438 |
| Compactness picked | 0.884746 | 0.902117 | 0.877321 | 0.045678 | 0.048344 | 0.068599 |
| Convexity non-picked | 0.949289 | 0.958056 | 0.977172 | 0.026361 | 0.023949 | 0.005872 |
| Convexity picked | 0.9645 | 0.972161 | 0.963824 | 0.017234 | 0.015915 | 0.026259 |
| EquivalentDiameter non-picked | 243.2795 | 255.0806 | 295.1151 | 20.87551 | 32.71105 | 40.18882 |
| EquivalentDiameter picked | 241.3765 | 275.9459 | 310.6608 | 21.88795 | 21.6528 | 36.09088 |
| Perimeter non-picked | 838.6234 | 867.0673 | 969.1439 | 88.94137 | 141.327 | 132.0822 |
| Perimeter picked | 807.7343 | 913.901 | 1045.665 | 82.91291 | 75.11183 | 136.2034 |
| Solidity non-picked | 0.95603 | 0.965228 | 0.978064 | 0.01263 | 0.011582 | 0.005312 |
| Solidity picked | 0.970506 | 0.973981 | 0.971821 | 0.010314 | 0.010764 | 0.01175 |
| Sphericity non-picked | 0.913856 | 0.928714 | 0.956602 | 0.036019 | 0.034792 | 0.009085 |
| Sphericity picked | 0.940291 | 0.949442 | 0.93593 | 0.024468 | 0.026003 | 0.036835 |
| Volume non-picked | 7281804 | 8618729 | 13584899 | 2031243 | 3524041 | 5595059 |
| Volume picked | 7154047 | 10666493 | 14930339 | 1966125 | 2617721 | 5220439 |
| Huh-7D12 | Avarage values | | | Deviance values | | |
|  | 0h | 24h | 48h | 0h | 24h | 48h |
| Area non-picked | 61673.07 | 70227.51 | 77159.74 | 4927.129 | 5841.263 | 11219.59 |
| Area picked | 49439.74 | 57233.46 | 67171.69 | 15242.78 | 13226.22 | 32314.72 |
| Circularity non-picked | 0.962427 | 0.936454 | 0.930264 | 0.017585 | 0.012617 | 0.014757 |
| Circularity picked | 0.947786 | 0.903583 | 0.936951 | 0.048818 | 0.088093 | 0.02155 |
| Compactness non-picked | 0.89938 | 0.838303 | 0.802588 | 0.057463 | 0.030124 | 0.057167 |
| Compactness picked | 0.912852 | 0.758813 | 0.859282 | 0.079562 | 0.183892 | 0.048782 |
| Convexity non-picked | 0.966077 | 0.94601 | 0.928114 | 0.02552 | 0.015448 | 0.02791 |
| Convexity picked | 0.980195 | 0.903425 | 0.957196 | 0.019923 | 0.094382 | 0.016818 |
| EquivalentDiameter non-picked | 279.9953 | 298.761 | 312.5677 | 11.27866 | 12.58281 | 23.32962 |
| EquivalentDiameter picked | 247.421 | 268.1606 | 283.7985 | 41.61129 | 31.01291 | 70.59777 |
| Perimeter non-picked | 929.0826 | 1026.187 | 1097.942 | 54.58214 | 57.51259 | 90.76403 |
| Perimeter picked | 812.8066 | 1005.42 | 963.5275 | 121.15 | 219.9312 | 244.3607 |
| Solidity non-picked | 0.972396 | 0.956485 | 0.951317 | 0.009129 | 0.00778 | 0.012402 |
| Solidity picked | 0.968258 | 0.928768 | 0.961115 | 0.028159 | 0.070038 | 0.012087 |
| Sphericity non-picked | 0.947853 | 0.915442 | 0.895293 | 0.030907 | 0.016402 | 0.032241 |
| Sphericity picked | 0.954439 | 0.86219 | 0.926617 | 0.043584 | 0.124268 | 0.025756 |
| Volume non-picked | 11086127 | 13609264 | 15761742 | 1264150 | 1819430 | 3401601 |
| Volume picked | 8331074 | 9871100 | 13170069 | 3584078 | 3666045 | 8622482 |
| 5-8F | Avarage values | | | Deviance values | | |
|  | 0h | 24h | 48h | 0h | 24h | 48h |
| Area non-picked | 51942.1 | 78253.65 | 113080.2 | 3351.208 | 9376.213 | 10937.97 |
| Area picked | 46536.8 | 79966.42 | 122583.7 | 11670.17 | 16822.67 | 16908.07 |
| Circularity non-picked | 0.952032 | 0.933539 | 0.939191 | 0.008034 | 0.010018 | 0.016303 |
| Circularity picked | 0.947844 | 0.924433 | 0.934102 | 0.017133 | 0.018956 | 0.016845 |
| Compactness non-picked | 0.846646 | 0.766136 | 0.757022 | 0.031801 | 0.044016 | 0.096875 |
| Compactness picked | 0.847969 | 0.724119 | 0.784488 | 0.060622 | 0.051744 | 0.026649 |
| Convexity non-picked | 0.94285 | 0.905487 | 0.895478 | 0.015937 | 0.023172 | 0.051907 |
| Convexity picked | 0.945074 | 0.884414 | 0.916323 | 0.027096 | 0.026608 | 0.013065 |
| EquivalentDiameter non-picked | 257.0303 | 315.0962 | 378.9911 | 8.376292 | 18.70962 | 18.54655 |
| EquivalentDiameter picked | 241.4343 | 317.1845 | 394.0034 | 31.0157 | 34.79115 | 28.97803 |
| Perimeter non-picked | 878.2387 | 1132.773 | 1376.169 | 34.03329 | 82.24547 | 97.37484 |
| Perimeter picked | 825.8193 | 1173.563 | 1398.243 | 114.4269 | 136.0343 | 109.3159 |
| Solidity non-picked | 0.964911 | 0.949935 | 0.953516 | 0.004639 | 0.007689 | 0.013536 |
| Solidity picked | 0.960139 | 0.94292 | 0.956135 | 0.011999 | 0.010948 | 0.0044 |
| Sphericity non-picked | 0.919972 | 0.874931 | 0.868197 | 0.017263 | 0.025154 | 0.057062 |
| Sphericity picked | 0.920251 | 0.850409 | 0.885584 | 0.033275 | 0.030402 | 0.015167 |
| Volume non-picked | 8506132 | 16171812 | 28035655 | 921241.3 | 2945436 | 4002430 |
| Volume picked | 7461751 | 16932902 | 31182759 | 2710532 | 5070299 | 6653687 |

|  | T47-D before picking | | T47-D after picking | |
| --- | --- | --- | --- | --- |
|  | average | deviance | average | deviance |
| Area | 45700.73929 | 5766.299399 | 45393.50896 | 7109.152976 |
| Circularity | 0.956389311 | 0.015729 | 0.968145424 | 0.006311041 |
| Compactness | 0.888773953 | 0.053685446 | 0.926342003 | 0.017675877 |
| Convexity | 0.963409773 | 0.022046834 | 0.978120421 | 0.007425406 |
| EquivalentDiameter | 240.7466932 | 15.13334803 | 239.7517183 | 17.77425783 |
| FeretDiameterMax | 261.412866 | 15.50735579 | 258.9882291 | 17.22651415 |
| FeretDiameterMaxOrthogonalDistance | 239.0201186 | 22.48833021 | 240.6096953 | 21.80733202 |
| FeretDiameterMin | 230.6537966 | 17.41509856 | 231.7962433 | 18.55491496 |
| LengthMajorDiameterThroughCentroid | 256.3113272 | 15.28633329 | 254.2189188 | 17.5878581 |
| LengthMinorDiameterThroughCentroid | 221.0002788 | 17.34987137 | 221.6759451 | 18.86293273 |
| Perimeter | 803.6329091 | 60.50532008 | 782.7745455 | 58.43719928 |
| Solidity | 0.969458453 | 0.011050636 | 0.977731819 | 0.004378111 |
| Sphericity | 0.94230522 | 0.028893342 | 0.96242223 | 0.009244178 |
| Volume | 7077943.441 | 1461607.919 | 7037093.813 | 1830118.299 |
|  | HUH-7D12 before | | HUH-after picking | |
|  | average | deviance | average | deviance |
| Area | 61673.07493 | 4927.129214 | 60076.27941 | 5635.707454 |
| Circularity | 0.962426801 | 0.01758487 | 0.967323842 | 0.016808041 |
| Compactness | 0.899379709 | 0.05746333 | 0.934096759 | 0.032553977 |
| Convexity | 0.966076681 | 0.025520436 | 0.982491883 | 0.010078754 |
| EquivalentDiameter | 279.9953386 | 11.27866077 | 276.2612234 | 13.08553022 |
| FeretDiameterMax | 303.3720268 | 19.9556144 | 297.9460246 | 13.13554044 |
| FeretDiameterMaxOrthogonalDistance | 278.7734168 | 10.20505336 | 272.0740872 | 16.31561066 |
| FeretDiameterMin | 269.9024897 | 9.665341031 | 265.6495593 | 14.19871241 |
| LengthMajorDiameterThroughCentroid | 296.0979137 | 15.83929128 | 290.1113952 | 14.17893058 |
| LengthMinorDiameterThroughCentroid | 259.3257633 | 9.027484687 | 255.6578191 | 15.34078391 |
| Perimeter | 929.0825556 | 54.58213733 | 898.0463 | 39.55802185 |
| Solidity | 0.972395819 | 0.009129401 | 0.978443023 | 0.006966394 |
| Sphericity | 0.947852553 | 0.030907056 | 0.966335578 | 0.017097088 |
| Volume | 11086127.01 | 1264149.627 | 10673615.83 | 1594862.042 |
|  | 5-8F before picking | | 5-8F after picking | |
|  | average | deviance | average | deviance |
| Area | 48339.12891 | 6150.572066 | 49332.38616 | 6965.524457 |
| Circularity | 0.948538523 | 0.009522291 | 0.942299111 | 0.015673743 |
| Compactness | 0.857761582 | 0.026914052 | 0.851423821 | 0.040779928 |
| Convexity | 0.950806698 | 0.011444281 | 0.950228622 | 0.016997708 |
| EquivalentDiameter | 247.5816786 | 15.83044309 | 249.9847371 | 17.87669154 |
| FeretDiameterMax | 269.6794232 | 22.38431504 | 276.7972591 | 19.70943744 |
| FeretDiameterMaxOrthogonalDistance | 251.4876863 | 15.37990052 | 250.82218 | 24.2985266 |
| FeretDiameterMin | 240.3612503 | 15.72895593 | 241.5237597 | 19.88890719 |
| LengthMajorDiameterThroughCentroid | 265.0936728 | 22.38348463 | 269.0394093 | 18.69799891 |
| LengthMinorDiameterThroughCentroid | 226.8609335 | 15.54178945 | 229.6124634 | 19.40579535 |
| Perimeter | 840.1702 | 57.03675707 | 852.6735 | 72.58626707 |
| Solidity | 0.962041506 | 0.006781886 | 0.961462267 | 0.006360445 |
| Sphericity | 0.926041033 | 0.01447716 | 0.922460805 | 0.022133356 |
| Volume | 7684312.415 | 1422977.924 | 7899610.291 | 1705070.218 |

**Supplementary Table 1. :** Morphological parameters of the living spheroids generated from the T-47D, Huh-7D12 and 5-8F carcinoma cell lines. Data were obtained through AnaSP analysis of the brightfield images acquired both in the long-term and short-term experiments.
